# Divergent adaptations of leaf functional traits to light intensity across common urban plant species in Lanzhou, northwestern China

**DOI:** 10.3389/fpls.2023.1000647

**Published:** 2023-01-25

**Authors:** Ketong Yang, Guopeng Chen, Junren Xian, Hailong Chang

**Affiliations:** ^1^ College of Forestry, Gansu Agricultural University, Lanzhou, China; ^2^ College of Environmental Sciences, Sichuan Agricultural University, Chendu, China; ^3^ College of Land Resources and Environment, Jiangxi Agricultural University, Nanchang, China

**Keywords:** light environment, relative benefit, scaling relationship, phenotypic plasticity, stoichiometry, urban plants

## Abstract

Leaves are the most important photosynthetic organs in plants. Understanding the growth strategy of leaves in different habitats is crucial for elucidating the mechanisms underlying plant response and adaptation to the environment change. This study investigated the scaling relationships of the laminar area (LA), leaf fresh mass (LFM), leaf dry mass (LDM), and explored leaf nitrogen (N) and phosphorus (P) content in leaves, and the relative benefits of these pairwise traits in three common urban plants (*Yulania denudata*, *Parthenocissus quinquefolia*, and *Wisteria sinensis*) under different light conditions, including (full-sun and canopy-shade). The results showed that: the scaling exponent of LDM vs LA (> 1, *p* < 0.05) meant that the LDM increased faster than LA, and supported the hypothesis of diminishing returns. The LFM and LDM had isometric relationships in all the three species, suggesting that the leaf water content of the leaves was nearly unaltered during laminar growth. *Y. denudata* and *W. sinensis* had higher relative benefit in full-sun habitats, while the reverse was observed in *P. quinquefolia*. The N and P content and the N:P ratio in full-sun leaves were generally higher than those of canopy-shade leaves. The leaves of the three urban plants exhibited a shift in strategy during transfer from the canopy shaded to the sunny habitat for adapting to the lower light conditions. The response of plant leaves to the environment shapes the rich variations at the leaf level, and quantification of the relative benefits of plants in different habitats provides novel insights into the response and adaptation strategies of plants.

## 1 Introduction

Leaves represent the primary light harvesting organs in vascular plants, and are the main components responsible for energy transfer between plants and the external environment (Milla and Reich, 2007; Sun et al., 2017). It is therefore important to understand how plants adapt to environmental variations by adjustment and complementation for ensuring survival and seeking maximum benefits. Despite differences, leaf sizes, laminar area (LA), and mass of lamina are closely related to each other (Wright et al., 2004; Li et al., 2008; Shi et al., 2020). Therefore, analyses of the relationship between LA and mass of lamina can aid in effective evaluation of the photosynthetic capacity, terrestrial biomass, and even ecosystem functions (Zhang and Feng, 2004; Niklas et al., 2007).

In scaling relationships, any two traits can be expressed by the scaling equation *y* = *β x ^α^*, where *x* and *y* are pairwise traits, *β* is the scaling (normalization) constant, and *α* is the scaling exponent (Niklas et al., 2007; Sun et al., 2017). In practical applications, this power function is often transformed to a logarithmic function, in which case the scaling equation takes the form log *y* = log *β* + *α* log *x* (Milla and Reich, 2007; Cheng et al., 2009). In this context, the hypothesis of diminishing returns predicts that the relationship between LA and laminar mass is greater than unity (that is, *α* > 1), indicating that despite incremental gains in laminar mass, the LA does not increase proportionally (Huang et al., 2019; Shi et al., 2020). If this hypothesis is true, the specific leaf area (SLA), which is a crucial index of photosynthetic efficiency and determined using the equation SLA = LA/leaf dry mass (LDM), would decrease with increasing LA.

In previous studies, researchers have often considered LA and LDM as benefits and costs, respectively, and they can calculate SLA (Milla and Reich, 2007; Li et al., 2008; Dinesh et al., 2019). Numerous empirical studies have demonstrated that specific areas of leaves are positively correlated with the net photosynthetic rate (Niklas et al., 2007), and the underlying message here is that LA and LDM are closely related to photosynthesis (Huang et al., 2019; Shi et al., 2020). As aforementioned, the formula can be used for theoretically determining the fresh and dry mass of laminae, and the relationship between the dry mass and fresh mass reflects the dynamic characteristics of the water content of leaves. Therefore, elucidating the complex relationship among laminar fresh mass, dry mass, and area by analyzing their scaling relationship is important for understanding the structural relationship of leaves.

When exposed to habitat stress, plants frequently employ phenotypic plasticity for adapting to the environment (Lusk, 2004; Kelsey and Jason, 2018; Esperón et al., 2020). When the levels of stress are extremely high, plants may induce a shift in ecological strategy, as described in the leaf economics spectrum (LES) (Wright et al., 2004). Apart from leaf morphology, alterations are also observed in leaf inclusions (water content or enzyme activity, for instance), including the LA, nitrogen (N) and phosphorus (P) content, and other parameters, in heterogeneous habitats (Liu et al., 2007; Dinesh et al., 2019). Light is an abiotic factor that is necessary for the survival of plants, and alterations in the intensity of light affect the normal growth and photosynthetic rate of plants, especially for understory species (Aleric and Kirkman, 2005; Selaya et al., 2008; Valladares and Niinemets, 2008; Liu et al., 2016). A previous study demonstrated that the presence of shade significantly decreases leaf size and laminar mass per unit area (reciprocal of SLA) in rainforest plants (Meng et al., 2014). Some other studies have confirmed that nitrogen concentration per unit leaf area was greater in current-year foliage from high-light environments than in current-year foliage from low-light environments (Zhang and Feng, 2004; Katahata et al., 2007). Unfortunately, quantitative comparisons are not effective in clarifying whether there has been a shift in plant adaptation strategies. It is therefore necessary to determine whether plants gain more relative benefits in shaded or unshaded habitats, and whether the alterations in different functional traits result from phenotypic plasticity or a shift in ecological strategy.

To this end, we selected three urban plants, namely, *Yulania denudata*, *Parthenocissus quinquefolia*, and *Wisteria sinensis*, and determined seven major functional traits of the leaves under different light intensities (full sun and canopy shade). We subsequently calculated the relative benefits in the different habitats, and proposed the following hypotheses: (1) the scaling relationship between the LA and laminar mass supports the hypothesis of diminishing returns; and (2) the adaptation strategy of leaves shifted under different conditions of light.

## 2 Materials and methods

### 2.1 Study site

The study was conducted in and around the campus of Gansu Agricultural University, Lanzhou, China. Lanzhou city is located in the north-west of the Loess plateau, and has a dominant subtropical continental monsoon climate with four distinct seasons. Summer is hot and rainy, with rainfall accounting for more than 60% of the year, while winter is cold and dry. The lowest and highest temperatures are -10°C (in February) and 36°C (in July), respectively. The annual mean temperature is 10.3°C and the annual mean precipitation is 357 mm. The soil types are primarily calcareous, chestnut, and cinnamon, and are nutrient barren with scarce natural vegetation.

### 2.2 Plant material

Urban plants are the main vegetation cover in cities, which are watered on a regular basis every month. In this study, we selected three common plants, namely, *Y. denudata*, *P. quinquefolia*, and *W. sinensis*. *Y. denudata* is a deciduous tree, *P. quinquefolia* and *W. sinensis* are deciduous lianas. Some of them were growing under the canopy of large trees or on the north side of tall buildings, while some were completely exposed to the sun. Based on the differences in light exposure, the habitats were classified as shaded and unshaded, or low light and high light habitats (**Table 1**).

**Table 1:** Light intensity of urban plants in different habitats.

Plant species	Light intensity	Mean ± SD (Lux)
*Y. denudata*	Low	4088.80 ± 968.40**^a^**
	High	100319.07 ± 3585.41**^b^**
*P. quinquefolia*	Low	3012.80 ± 215.20**^a^**
	High	102865.60 ± 6670.33**^b^**
*W. sinensis*	Low	2080.27 ± 1150.53**^a^**
	High	106990.27 ± 4926.55**^b^**

The different lowercase letters indicate significant differences at the 0.05 level.

The sampling was conducted in the month of July in 2021. A total of 20 individuals (10 individuals each under low and high light) were selected for each of the three species, and at least 80~150 leaves were collected from different habitats. The leaves were collected at the height of 1.8 m and the light intensity at the corresponding position was simultaneously measured with a handheld illuminometer (TES-1334A, Taiwan, China). All the collected leaves were subsequently placed in plastic self-sealing bags in a portable incubator with ice bags for preventing the leaf blades from deforming and losing water. The leaf samples were then transferred to the laboratory for subsequent measurements.

### 2.3 Leaf traits measurement

The collected leaf samples were individually scanned using a scanner (EPSON V39, Indonesia), and the images were saved in bitmap format at a resolution of 300 dpi. The ImageJ software (version 1.48, National Institutes of Mental Health, Maryland, America) was used to generate the leaf profiles as black and white images. The length, width, and area (LA, cm^2^) of the leaf blades were measured using the ImageJ software. The leaf fresh mass (LFM, g) was determined, following which the leaves were dried in a ventilated oven (Taisite, WHL45B, Tianjin, China) at 105°C for 20 min, and the temperature was reduced to 75°C until a constant dry mass was reached, which represented the leaf dry mass (LDM, g) (Pérez et al., 2013; Huang et al., 2019). The LFM and LDM were measured using an electronic balance (0.0001 g, Zhuojing Experimental Equipment Co. Ltd., BMS, Shanghai, China). Specific leaf area (SLA, cm^2^/g) was calculated by LA/LDM. The dried leaves were finally crushed and passed through a fine 800 mesh sieve for analyzing the total N and P content. The total N content was determined by the H_2_SO_4_-H_2_O_2_ method, and the total P was determined using the molybdenum antimony scandium colorimetric method. Finally, the phenotypic plasticity index (*PPI*) was calculated according to Valladares et al. (2006) method, i.e. *PPI* = [(max - min)/max], and it was a dimensionless parameter, the greater value indicated stronger phenotypic plasticity.

### 2.4 Scaling relationship analysis

The relationships among LA, LFM, and LDM (namely, LDM vs. LFM, LFM vs. LA, and LDM vs. LA) can be described by a mathematical equation of the type *y* = *β x ^α^*, which can be linearized as log (*y*) = log (*β*) + *α* log (*x*), where *x* and *y* determine whether the relationship is isometric (*α* = 1) or allometric (*α* > 1 or *α* < 1). The *β* term represents the y-intercept of the relationship, and its value does not determine the nature of the relationship. If two lines with the same slope are compared, the difference between their respective *β* values indicates the difference between the indices. The 95% confidence intervals (95% CI) of *α* and *β* were calculated by Standardized Major Axis (SMA) regression (i.e. Model type II) using the Standardized Major Axis Tests and Routines (SMATR) software, version 2.0 (Falster et al., 2006). In order to make the data more closely to the normal distribution, the leaf trait values were analyzed by scaling after lg10 conversion. Additionally, the significant difference between the slope and unity was calculated for all the parameters for assessing the relationship between the allometric growth index and unity by Wald significance test (Warton et al., 2006). If the difference between the slope and unity was not significant, the relationship between the two indices was considered to be isometric; however, if the slope was greater than or less than unity, the relationship between the two indices was considered to be allometric.

### 2.5 Relative benefit analysis

The trade-offs between pairwise traits (A vs B) were calculated as described hereafter. The benefit for a single object (A or B) is defined as the relative deviation from the mean for a given observation. Given the observations for an individual object A, the magnitude of benefit for object A (B_A_, A/B) is calculated as: B_A_ = (A_OBS_-A_min_)/(A_max_-A_min_) (Bradford and D’Amato, 2012), where A_OBS_ represents the observed value of A/B, while A_max_ and A_min_ are calculated from all the observed values of A/B (Sun and Wang, 2016). The trade-offs range from 0 to 1, and can be conceptualized as the proportion of possible benefits in object A (A/B). In cases where certain objects are considered to be more valuable or important than others, individual objects (A/B) can be weighted for incorporating these differences during the calculation of overall benefits and trade-offs. A simple strategy for quantifying the magnitude of the trade-offs between A and B involves calculating the root mean square error (RMSE) of the individual benefits of A or B (Lu et al., 2014; Sun and Wang, 2016). The RMSE approximates the average deviation from the mean benefit, and is calculated in two dimensions by determining the distance between the coordinates of the paired traits and the “1:1 line” where the trade-off is zero (Bradford and D’Amato, 2012). This method represents an effective strategy for quantifying the relationship between A and B. A preliminary definition of relative benefit has been provided by Bradford and D’Amato (2012), and studies by Lu et al. (2014) and Sun and Wang (2016) have provided detailed descriptions.

### 2.6 Statistical analysis

The differences in the N and P contents and the N:P ratio across the different habitats were determined by one-way analyses of variance (ANOVA). Light habitats and species difference and their interaction were conducted by two-ways ANOVA analysis. Principal component analysis (PCA) was performed for investigating the shifts in adaptation strategy across different conditions of light. Data analyses were performed using SPSS 20.0 (Chicago, IBM Corp, USA), and the graphs were prepared using Origin 2019 (https://www.originlab.com). The measurements obtained for the different parameters are presented as the mean ± standard deviation (SD).

## 3 Results

### 3.1 Variations in leaf traits and *PPI* under different light habitats

The intensity of light significantly altered all the leaf traits with the exception of the LFM of *P. quinquefolia* and the LA of *W. sinensis*. The LFM, LDM, and LA of *Y. denudata* were 1.48 ± 0.47 g, 0.33 ± 0.11 g, and 76.67 ± 20.05 cm^2^, respectively, under conditions of low light (**Table 2**); which significantly decreased to 0.91 ± 0.24 g, 0.26 ± 0.07 g, and 41.45 ± 10.01 cm^2^, respectively, as the intensity of light increased. The LDM and LA of *P. quinquefolia* were 0.19 ± 0.07 g and 59.75 ± 19.70 cm^2^, respectively, under conditions of low light; which changed to 0.23 ± 0.10 g and 47.58 ± 16.27 cm^2^, respectively, when the intensity of light increased, while the LFM remained unaltered. The LFM and LDM of *W. sinensis* increased significantly from 0.31 ± 0.18 g and 0.08 ± 0.05 g, respectively, to 0.45 ± 0.16 g and 0.17 ± 0.06 g, respectively, as the intensity of light increased, while the LA remained unaltered. For the three urban plants, the SLA in low light habitat was significantly larger than that in high light habitat (*p*<0.05). Interestingly, we observed that the *PPI* of *Y. denudata* and *P. quinquefolia* for the leaf traits were 0.52–0.77 and 0.54–0.84, respectively, under conditions of low light; and were 0.41–0.77 and 0.70–0.91, respectively, under conditions of high light. However, the *PPI* of *W. sinensis* was higher under low light condition (0.49–0.93) and lower (0.54–0.80) under high light condition. We found that the *PPI* of SLA was always the smallest for all kinds of plants and light habitats.

**Table 2:** The difference and phenotypic plasticity index (*PPI*) of urban plant leaf traits in different light habitats.

Plant species	Traits	Low light		High light	
		Mean ± SD	*PPI*	Mean ± SD	*PPI*
*Y. denudata*	LFM (g)	1.48 ± 0.47	0.73	0.91 ± 0.24 *	0.77
	LDM (g)	0.33 ± 0.11	0.77	0.26 ± 0.07 *	0.77
	LA (cm^2^)	76.67 ± 20.05	0.64	41.45 ± 10.01 *	0.71
	SLA (cm^2^/g)	236.47 ± 36.37	0.52	160.54 ± 16.37 *	0.41
*P. quinquefolia*	LFM (g)	0.89 ± 0.34	0.83	0.89 ± 0.39 **^ns^**	0.90
	LDM (g)	0.19 ± 0.07	0.84	0.23 ± 0.10 *	0.91
	LA (cm^2^)	59.75 ± 19.70	0.77	47.58 ± 16.27 *	0.83
	SLA (cm^2^/g)	325.58 ± 47.73	0.54	219.35 ± 34.49 *	0.70
*W. sinensis*	LFM (g)	0.31 ± 0.18	0.92	0.45 ± 0.16 *	0.79
	LDM (g)	0.08 ± 0.05	0.93	0.17 ± 0.06 *	0.80
	LA (cm^2^)	30.88 ± 16.47	0.90	28.63 ± 9.23 **^ns^**	0.78
	SLA (cm^2^/g)	399.81 ± 46.82	0.49	171.51 ± 21.39 *	0.54

ns indicated no significant difference in leaf trait values under different light habitats, * indicated significant difference at the 0.05 level.

### 3.2 Scaling relationship between leaf traits and their relative benefits

**Figure 1:**
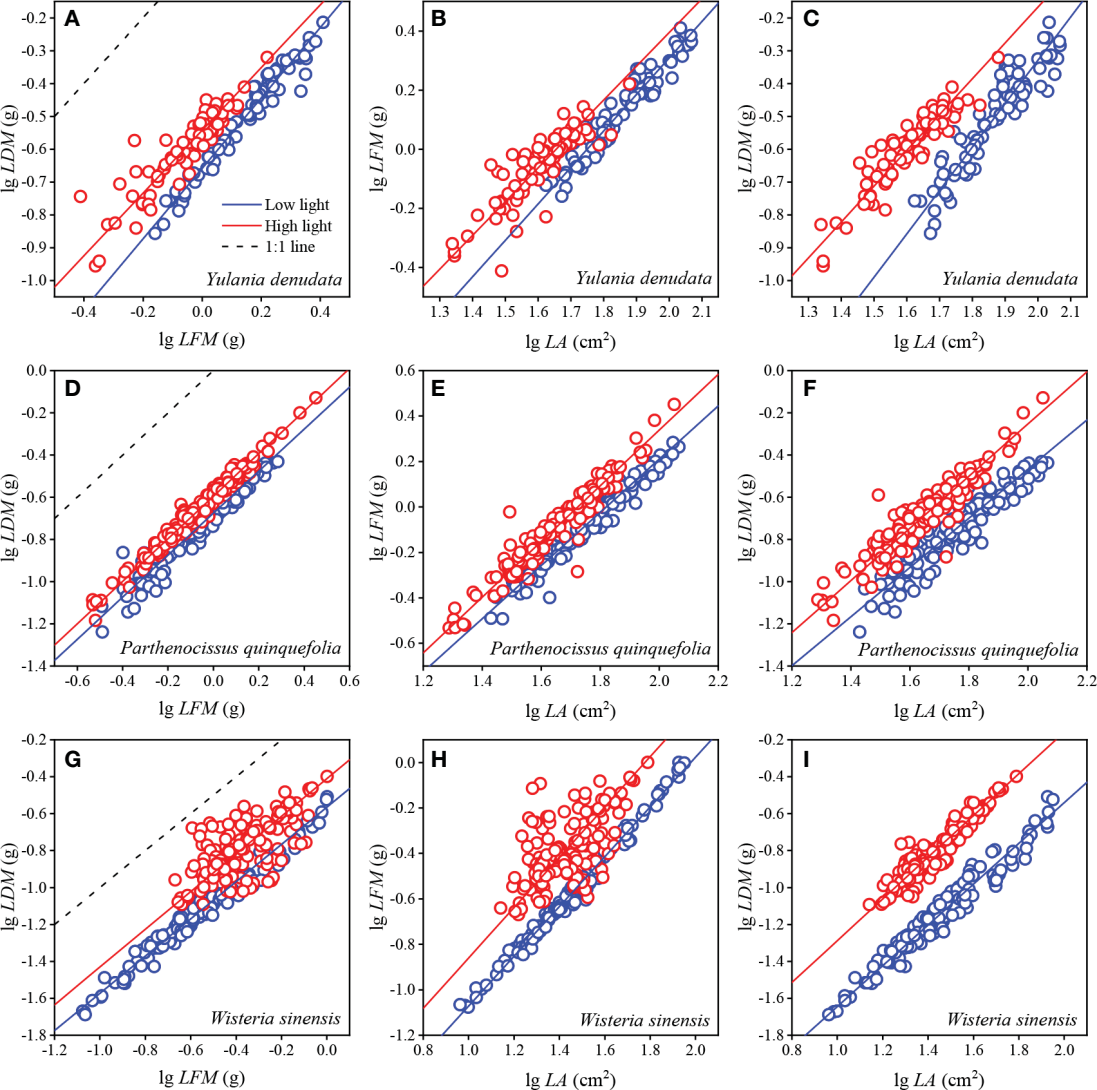
Scaling relationships between leaf traits in different urban plants. **(A–C)** represent the fitting relationship between *LDM* with *LFM*, *LFM* with *LA*, and *LDM* with *LA* of *Y. denudata* leaves, respectively. **(D–F)** represent the fitting relationship between *LDM* with *LFM*, *LFM* with *LA*, and *LDM* with *LA* of *P. quinquefolia* leaves, respectively. **(G–I)** represent the fitting relationship between *LDM* with *LFM*, *LFM* with *LA*, and *LDM* with *LA* of *W. sinensis* leaves, respectively. Circles represent the observed values; the blue and red solid lines represent SMA fitting lines between leaf traits under low and high light habitats, respectively; and dotted lines represent 1:1 line. Some 1:1 lines are not shown due to the proportional value of the axes.

**Figure 2:**
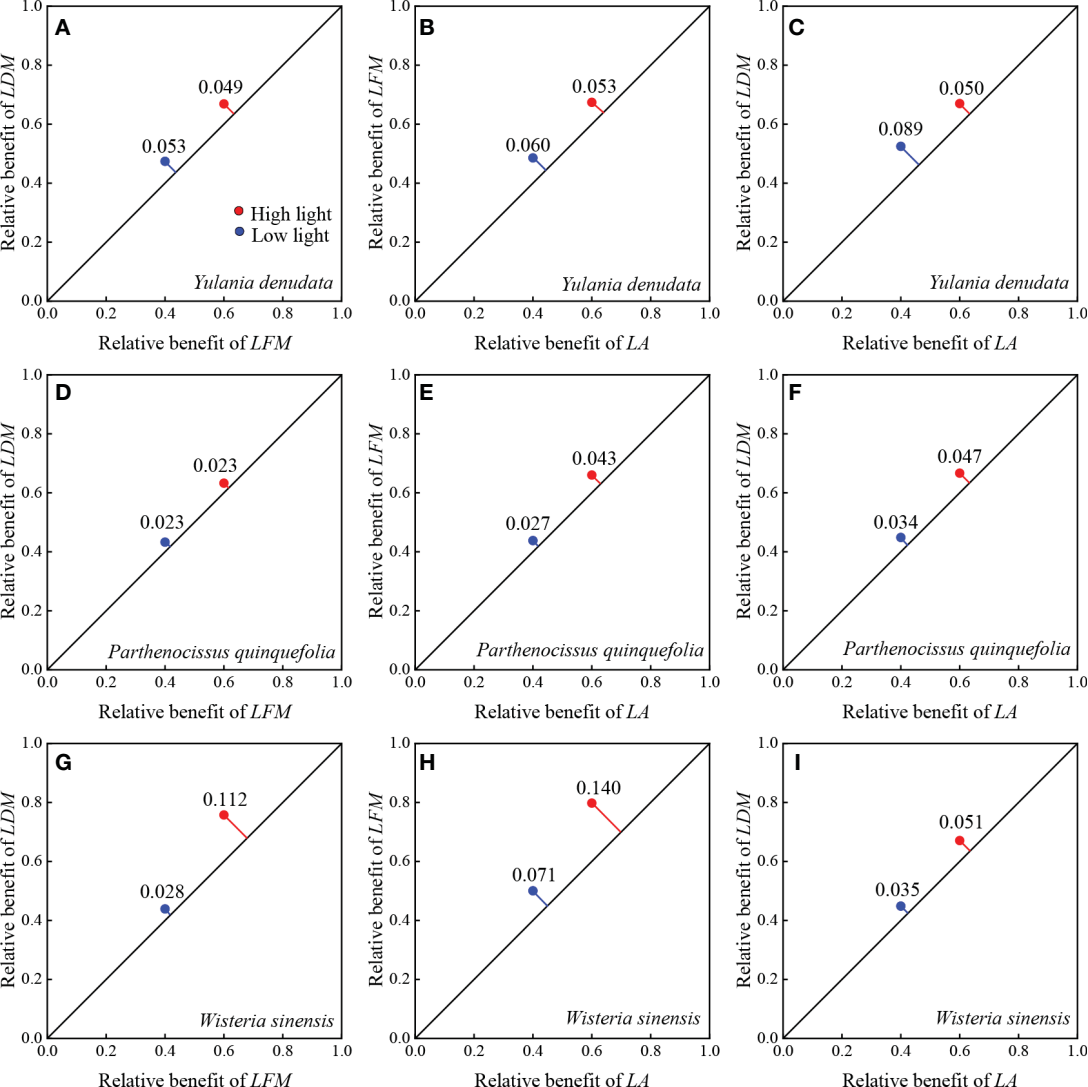
Relative benefits between paired leaf traits of different urban plant. **(A–C)** represent the relative benefit between *LDM* with *LFM*, *LFM* with *LA*, and *LDM* with *LA* of *Y. denudata* leaves, respectively. **(D–F)** represent the relative benefit between *LDM* with *LFM*, *LFM* with *LA*, and *LDM* with *LA* of *P. quinquefolia* leaves, respectively. **(G–I)** represent the relative benefit between *LDM* with *LFM*, *LFM* with *LA*, and *LDM* with *LA* of *W. sinensis* leaves, respectively. The blue and red points indicate low light and high light habitats, respectively. The relative benefit is represented by the RMSE of paired traits. The RMSE represents the distance from the coordinate of the paired traits to the diagonal 1:1 line where the trade-off is zero. The farther the distance, the larger the relative benefit.

For *Y. denudata*, the slopes between leaf traits were significantly higher than 1 (*p*<0.05) (**Figure 1**; **Table 3**), except that the slope of LDM vs LFM was not significant in the high light habitat (*p*=0.319). Similarly, the slope of LDM vs LFM for *P. quinquefolia* did not show markedly significant differences from unity under conditions of both low and high light habitats, while the slopes of the other pairwise traits, namely, LFM vs LA and LDM vs LA, were significantly different from unity (*p*<0.001). For *W. sinensis*, the slopes of only LDM vs LFM under both conditions of light, and LFM vs LA under conditions of low light were not statistically different from unity, while the slopes of the other pairwise traits were significantly different from unity (*p*<0.001). By summarizing the relative benefits for the three species, we observed that *Y. denudata* had higher relative benefits under low light habitat for all the pairwise traits (**Figure 2**), while the reverse was observed for *P. quinquefolia* and *W. sinensis*.

**Table 3:** Scaling relationships of leaf traits in urban plants under different light habitats.

Plant species	Traits	Habitats	*R* ^2^	*p*	Slope	Intercept	Isometric
					*α*	*β*	*p*
*Y. denudata*	LDM vs LFM	LL	0.96	< 0.01	1.07	-0.66	< 0.050
		HL	0.83	< 0.01	0.95	-0.54	0.319
	LFM vs LA	LL	0.92	< 0.01	1.24	-2.16	< 0.001
		HL	0.75	< 0.01	1.14	-1.89	< 0.050
	LDM vs LA	LL	0.86	< 0.01	1.32	-2.97	< 0.001
		HL	0.86	< 0.01	1.09	-2.35	< 0.050
*P. quinquefolia*	LDM vs LFM	LL	0.93	< 0.01	1.00	-0.68	0.850
		HL	0.97	< 0.01	1.01	-0.60	0.514
	LFM vs LA	LL	0.96	< 0.01	1.17	-2.13	< 0.001
		HL	0.92	< 0.01	1.23	-2.11	< 0.001
	LDM vs LA	LL	0.87	< 0.01	1.17	-2.80	< 0.001
		HL	0.89	< 0.01	1.24	-2.73	< 0.001
*W. sinensis*	LDM vs LFM	LL	0.96	< 0.01	1.01	-0.56	0.608
		HL	0.49	< 0.01	1.02	-0.41	0.722
	LFM vs LA	LL	0.99	< 0.01	1.10	-2.17	< 0.001
		HL	0.41	< 0.01	1.11	-1.97	0.111
	LDM vs LA	LL	0.95	< 0.01	1.11	-2.75	< 0.001
		HL	0.88	< 0.01	1.13	-2.42	< 0.001

LFM and LDM are represented in g; LA is represented in cm^2^. LL, low light; HL, high light. α is the slope or the scaling exponent; β is the intercept or scaling constant; the first p indicates linear correlation; the isometric p indicates whether the slope is significantly different from 1, and p < 0.05 indicates that the slope is significantly different from 1.

### 3.3 N and P content of leaves under different light habitats

**Figure 3:**
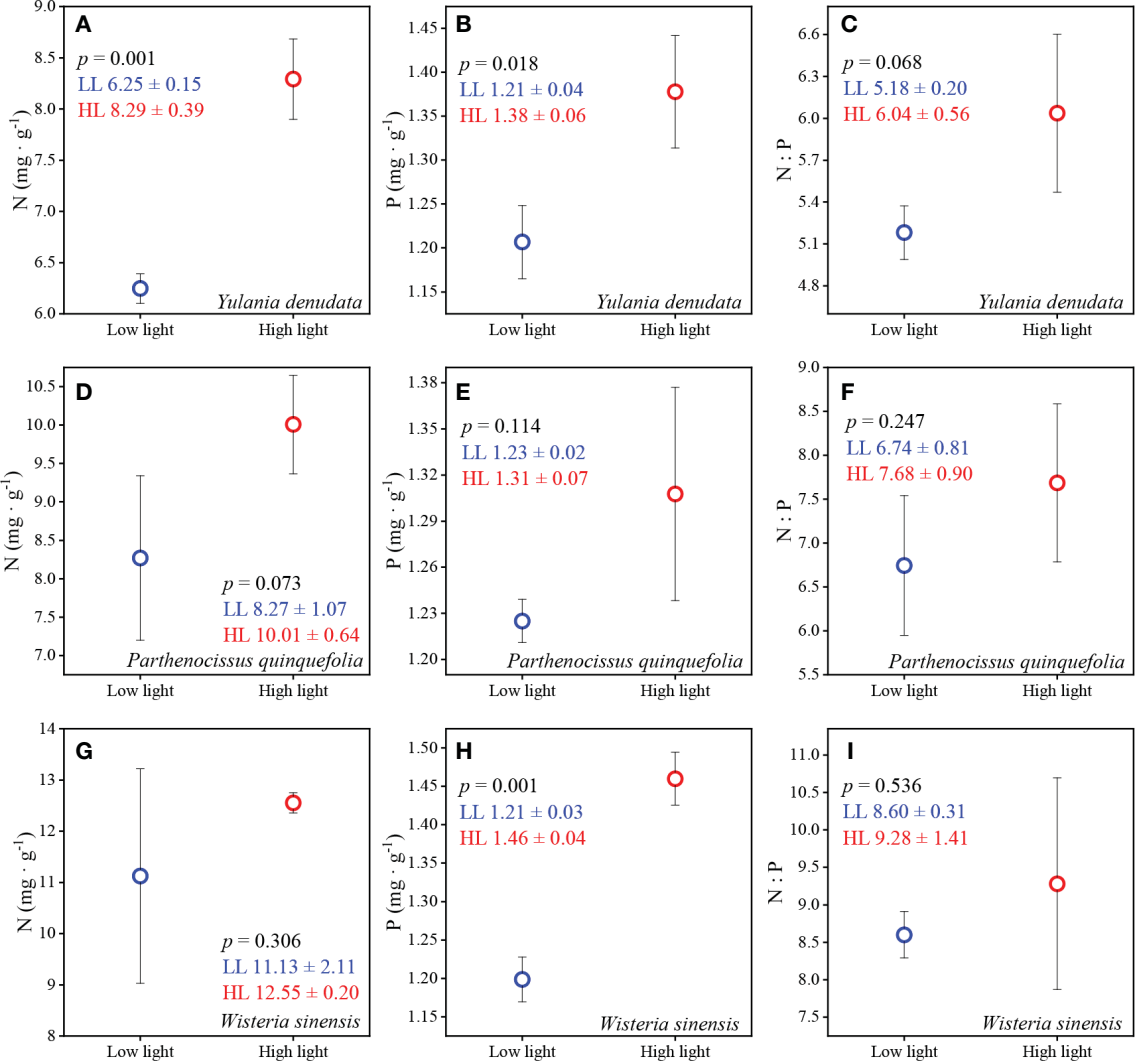
The N and P contents and N:P ratios under conditions of different light habitats. **(A–C)** represent the N, P content and N:P of *Y. denudata* leaves under different light habitats, respectively. **(D–F)** represent the N, P content and N:P of *P. quinquefolia* leaves under different light habitats, respectively. **(G–I)** represent the N, P content and N:P of *W. sinensis* leaves under different light habitats, respectively. The blue and red circles represent low light and high light, respectively. The numerical values in the figures represent the mean ± SD. *p* < 0.05 indicates a significant difference between the two light environments.

The plants that grew under high light intensity had higher N, P content and N:P ratios compared to low light intensity (**Figure 3**). The N and P contents of *Y. denudata* were 6.25 ± 0.15 mg·g^-1^ and 1.21 ± 0.04 mg·g^-1^, respectively, under low light conditions, and were significantly lower than those under high light intensity, which were 8.29 ± 0.39 mg·g^-1^ and 1.38 ± 0.06 mg·g^-1^, respectively. The N:P ratio under low and high light intensities were 5.18 ± 0.20 and 6.04 ± 0.56 (*p*=0.068), respectively. The N, P, and N:P ratio were 8.27 ± 1.07 mg·g^-1^, 1.23 ± 0.02 mg·g^-1^, and 6.74 ± 0.81, respectively, for *P. quinquefolia* under low light condition, and 10.01 ± 0.64 mg·g^-1^, 1.31 ± 0.07mg·g^-1^, and 7.68 ± 0.90, respectively, under high light intensity. *W. sinensis* had different N content and N:P ratios under different light intensities, which were 11.13 ± 2.11mg·g^-1^ and 8.60 ± 0.31, respectively, under high light intensity, and 12.55 ± 0.20 mg·g^-1^ and 9.28 ± 1.41, respectively, under low light intensity. The P content in the leaves of *W. sinensis* was high, being 1.46 ± 0.04mg·g^-1^, which was significantly higher than that under conditions of low light, which was 1.21 ± 0.03mg·g^-1^.

### 3.4 Combined effects of light and species on leaf traits

Two-ways ANOVA analysis showed that light habitats had significant effect on all traits except N:P (*F*=0.772, *p* > 0.05) (**Table 4**). Species differences had significant effects on all traits except LDM (*F*=2.799, *p* > 0.05). In addition, the interaction of the light habitats and species difference had significant impact on LFM, LDM, LA, SLA, and P content (*p* < 0.05). These results further supplemented and explained the results of one-way ANOVA analysis for element content.

**Table 4:** Results of two-ways ANOVA for the effects of light conditions, species and their interactions on functional traits of urban plants.

Morphological trait	Factor	*df*	LFM	LDM	LA	SLA
			*F*	*F*	*F*	*F*
	Light	1	37.854 ***	9.959 **	189.92 ***	2394.029 ***
	Species	2	438.488 ***	271.936 ***	248.150 ***	304.924 ***
	Light×Species	2	75.412 ***	59.954 ***	56.778 ***	300.128 ***
Chemical trait	Factor	*df*	N	P	N:P	
			*F*	*F*	*F*	
	Light	1	13.256 **	61.765 ***	0.772 ns	
	Species	2	30.919 ***	2.799 ns	20.714 **	
	Light×Species	2	0.141 ns	5.552 *	1.549 ns	

* p < 0.05; ** p < 0.01; *** p < 0.001; ns, no significant.

### 3.5 Shift in the adaptation strategies of leaves under different light habitats

**Figure 4:**
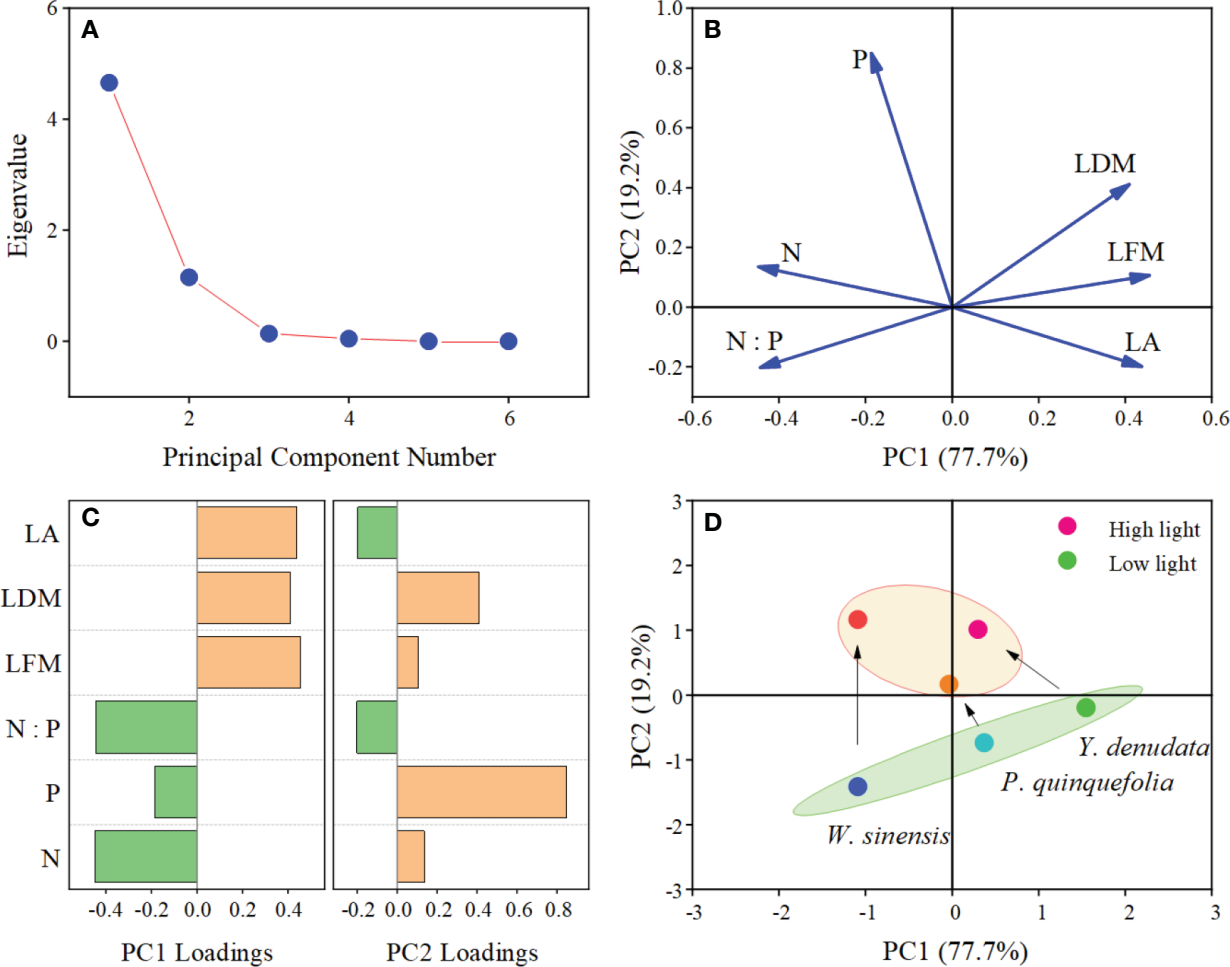
PCA analysis of leaf traits of urban plants in different light habitats. **(A)** Scree plot; **(B)** Loading plot; **(C)** Loadings of plant traits on the first and second axes; **(D)** Score plot and strategy shifts of the different plants under different light habitats.

PCA of the six leaf traits demonstrated that the explanatory rates of the first and second principal components were 77.7% and 19.2%, respectively (**Figure 4**). The absolute value of LFM, LDM, LA, N content, and N:P ratio were greater than 0.3 on the PC1 axis, while the absolute value of the LDM and P content were greater than 0.3 on PC2 axis, which indicated that these six traits can effectively explain the variation in leaf traits under different light habitats in different dimensions. Lastly, the results of PCA revealed an obvious shift in the adaptation strategy from a larger LA and lower N and P contents under low light habitat to a smaller LA and higher N and P contents under high light habitat in the three plants.

## 4 Discussion

Plants show different traits in different light habitats. Consistent with our hypothesis, light significantly changed leaf morphological traits and element content. The relationship between LDM and LA of all plants in different light habitats showed diminishing returns, that is, with the increase of leaf dry mass, the increase of leaf area gradually decreased. Except for *Y. denudata*, most LDM and LFM showed isomeric relationship, that is, leaf water content gradually increased with leaf mass. From low light to high light, the leaves tended to have higher P content. In conclusion, the plants growth strategies changed under different light habitats.

### 4.1 Scaling relationships between leaf traits and relative benefits

The scaling relationships of leaf traits has been confirmed by a number of empirical studies, which demonstrated that the habitat, developmental factors, and phylogeny affect the relationships between leaf traits (Niklas et al., 2007; Shi et al., 2020). A previous study by Sun et al. (2017) on five species of bamboos demonstrated a scaling relationship between LDM and LA under different conditions of light. Similarly, our results indicated that there was a scaling relationship between LDM and LA under different light condition, which supported the law of diminishing returns. Briefly, as the LA increased, the LDM increased more rapidly, which consequently decreased the SLA. Interestingly, the scaling exponent (*α*) of the LDM and LA of *Y. denudata* under low light (*α* = 1.32) was larger than that of high light intensity (*α* = 1.09), whereas the scaling exponents of *P. quinquefolia* (*α* = 1.17 and 1.24, respectively, under low and high light) and *W. sinensis* (*α* = 1.11 and 1.13, respectively, under low and high light) showed a reverse trend. We speculated that this could be attributed to the differences in plant life forms and leaf forms (for instance simple and compound leaves). *Y. denudata* is a small tree, while the other two plants are woody lianas. There are significant differences in the hydraulic structures of these plants, which may lead to differences in laminar and petiolar investment (Maréchaux et al., 2017). A considerable portion of the biomass of *Y. denudata* is contributed to strengthening the support system and petiolar transportation. In terms of leaf forms, both *P. quinquefolia* and *W. sinensis* have compound leaves, implying that apart from petioles, the laminar biomass of these plants is also invested in rachides (Xu et al., 2009). If a scaling relationship exists between petioles (rachides) and laminar biomass, plants with different leaf forms may have significant differences in the investment of laminar biomass (Yang et al., 2009).

In general, regions with high light intensity have lower water content in soils than regions with low light intensity; therefore, trade-offs between laminar water content and LDM during plant growth are more likely under high light intensity than under low light (Meng et al., 2014; Umaña and Swenson, 2019; Li et al., 2020). The relationship between LDM and LFM may reflect the variations in laminar water content, which is the basis of photosynthesis (Santiago et al, 2018; Dinesh et al., 2019). Our study also demonstrated the LDM and LFM increased at an equal rate (nearly 1:1) under almost all conditions, indicating that when the LFM increased, the LDM also increased gradually, while the water content of the leaves remained constant. These findings are inconsistent with the results of the study by Shi et al. (2020) on *Fallopia multiflora*. These variations are attributed to differences in the environment between these studies. Natural rainfall is the only source of water for wild plants; however, water is not a limiting factor for urban plants. Therefore, urban plants do not have a very strong demand for water storage during growth (Song et al., 2021).

Plant SLA in low light habitat was significantly larger than that in high light habitat, which was consistent with most other studies (Baird et al., 2017; Power et al., 2019). Plants in high light environments need to invest more in defense against heat and damage than shaded plants. Plants under shade may increase their LA to gain more light, which is consistent with the significant difference in LA between different light habitats. We also observed that the *PPI* of SLA was the smallest compared with other traits, which also indicated that SLA was stable and could be used to refer to the photosynthetic capacity of plants (Liu et al., 2016; Dinesh et al., 2019).

The trade-offs between pairwise traits in different habitats can more clearly illustrate the adaptation of plants to the environment. Only rarely studies have quantified plant habitat preferences using relative benefits (Cheng et al., 2021; Wang et al., 2021). In general, *P. quinquefolia* and *W. sinensis* have greater relative benefits under high light habitat, while *Y. denudata* have greater relative benefits under low light conditions. This indicates that *P. quinquefolia* and *W. sinensis* can maximize the advantages of functional traits under high light conditions, while the reverse is observed in *Y. denudata*. This may also be the underlying reason why *Y. denudata* has a higher slope in low light. In contrast, the slope of the *P. quinquefolia* and *W. sinensis* varied little in different light conditions.

### 4.2 Stoichiometric characteristics and strategy shift in leaves

The adaptation of plants to different habitats not only manifests in leaf morphology but is also observed in the alterations in leaf inclusions. The results of this study confirmed that the leaves of plants growing under conditions of low light had lower N and P contents and N:P ratios. However, this finding was not consistent with the results of the study by Niinemets et al. (2006), which reported that plants with larger SLA, corresponding to plants under conditions of low light in this study, have higher N and P content. Nevertheless, some studies have suggested that the N and P content of leaves are expected to increase with increasing light (Katahata et al., 2007; Puglielli et al., 2020). Compared to conditions of low light, high light conditions enhance photosynthesis to a certain extent, and also promote plant growth and development, thereby gradually increasing the N and P content in leaves. The N:P ratio of the three plants studied herein was less than 14 or 20 under different light conditions, indicating that the growth of these plants was restricted by N availability (Koerselman and Meuleman, 1996; Güsewell, 2004; Yan et al., 2017). The plants growing under conditions of low light were more severely restricted by N deficiency, compared to those growing under conditions of high light. We hypothesized that the lower light intensity restricted photosynthesis and leaf growth, and that the canopy intercepted most of the atmospheric N deposition in the soil. Low N deposition in the soil in which plants are growing can be an important factor limiting N uptake, especially for urban plants.

The relationships between leaf traits enhance plant adaptability to the heterogeneous environment. As predicted by the LES (Wright et al., 2004), the dimensions of LFM, LDM, and LA are different from the N, P contents and N:P ratios, and their coordination may ensure the normal growth of plants under different conditions of light (Baird et al., 2017; Puglielli et al., 2020). It is worth mentioning that the three urban plants studied herein employed adaptive strategy shifting in environments with different conditions of light. The relative displacement of *P. quinquefolia* was found to be minimal, suggesting that it has a higher tolerance to conditions of low light than *W. sinensis* and *Y. denudta*. While certain plants are naturally capable of tolerating shade, most plants require sufficient light for carbon gains. Among the six traits, P content appeared to be independent of the others, and the contribution of P to the PC2 axis was high. This is consistent with the result of significant difference of P content in leaves under different light habitats, and also suggests that future research should pay attention to the change of P content.

## 5 Conclusion

Adaptive strategy shifting is an important mechanism ensuring the survival of plants in heterogeneous environments. This study evaluated plant tendencies under different conditions of light by incorporating the relative benefits of pairwise leaf traits, and the results demonstrated that plants perform adaptative strategy shifting in environments under different conditions of light. In general, the relationship between LA and LDM under different conditions of light supported the law of diminishing returns without exception, and there were significant differences in the stoichiometric characteristics of leaves under different conditions of light. In future studies, the coupling relationship between leaf traits and elements should be clarified, and the adaptation of plants to habitat changes should be more fully understood in combination with plant biomass and root or other traits.

## Data availability statement

The data analyzed in this study is subject to the following licenses/restrictions. To obtain data, contact the corresponding author. Requests to access these datasets should be directed to KY, yangkt1996@126.com.

## Author contributions

KY and HC: methodology, formal analysis, visualization, writing-original draft. GC and JX: conceptualization, project administration. All authors contributed to the article and approved the submitted version.
